# Implementation of hyperspectral imaging in a trauma resuscitation room: a randomized controlled trial

**DOI:** 10.1186/s13049-022-01057-7

**Published:** 2022-12-09

**Authors:** Stephan Katzenschlager, Maximilian Dietrich, Franziska Peterstorfer, Katharina Manten, Maik von der Forst, Rouven Behnisch, Christine Leowardi, Alexander Studier-Fischer, Felix Nickel, Markus A. Weigand, Frank Weilbacher, Erik Popp

**Affiliations:** 1 Department of Anesthesiology, Heidelberg University Hospital, Im Neuenheimer Feld 420, 69120 Heidelberg, Germany; 2 Institute of Medical Biometry, Heidelberg University, Heidelberg, Germany; 3 Department of General, Visceral, and Transplantation Surgery, Heidelberg University Hospital, Heidelberg, Germany

**Keywords:** Trauma, Shock, Microcirculation, Hyperspectral imaging, Diagnostics

## Abstract

**Background:**

Hyperspectral imaging (HSI) is a novel imaging technology with the ability to assess microcirculatory impairment. We aimed to assess feasibility of performing HSI, a noninvasive, contactless method to assess microcirculatory alterations, during trauma resuscitation care.

**Methods:**

This randomized controlled clinical trial was conducted in a dedicated trauma resuscitation room of a level one trauma center. We included adult patients who were admitted to the trauma resuscitation room. Patients were allocated in a 1:1 ratio to the HSI group (intervention) or control group. In addition to the standard of care, patients in the intervention group had two hyperspectral recordings (HSR) of their hand palm taken. Primary outcomes were the treatment duration of the primary survey (until end of ABCDE-evaluation, ultrasound and evaluation by the trauma team) and the total resuscitation room care (until transport to definitive care) as well as the ability to perform measurements from all HSR. Secondary outcomes were analyses from the intervention group compared to HSI measurements of 26 healthy volunteers including an analysis based on the ISS (Injury severity score) (< 16 vs. ≥ 16). Care givers, and those assessing the outcomes were blinded to group assignment.

**Results:**

Our final analysis included 51 patients, with 25 and 26 allocated to the control and intervention group, respectively. There was a statistically significant shorter median duration of the primary survey in the control group (03:22 min [Q1–Q3 03:00–03:51]) compared to the intervention group (03:59 min [Q1–Q3 03:29–04:35]) with a difference of −37 s (95% CI −66 to −12). Total resuscitation room care was longer in the control group, but without significance: 60 s (95% CI −60 to 180). From 52 HSI, we were able to perform hyperspectral measurements on all images, with significant differences between injured patients and healthy volunteers.

**Conclusion:**

HSI proved to be feasible during resuscitation room care and can provide valuable information on the microcirculatory state.

*Trial registration* DRKS DRKS00024047–www.drks.de. Registered on 13th April 2021.

**Supplementary Information:**

The online version contains supplementary material available at 10.1186/s13049-022-01057-7.

## Introduction

### Background

Shock following major trauma is one of the leading causes of death following major trauma. The definition describes shock as tissue hypoxia due to insufficient blood flow to the body’s tissues. Current clinical practice lacks the possibility to directly measure microcirculatory blood flow in critically injured patients [1]. Clinical examination of tissue perfusion using capillary refill time (CRT) has been proven to be a fast and valuable tool and is well established in trauma care. However, CRT is subject to interobserver variability which can impact clinical treatment [2, 3]. Indirect microcirculatory parameters, such as lactate and (non-) invasive blood pressure, respectively, can only suggest a reduced microcirculatory state.

Hyperspectral imaging (HSI) is a novel technique in medical imaging. In previous publications it has already been shown that HSI has the ability to record organ-specific spectral reflectance [4] and that it has the ability to evaluate changes in microcircular capillary perfusion of tissue in porcine models [4]. It has now been used to assess the quality of microcirculatory perfusion in critically ill patients [5]. Hyperspectral measurements allow a comprehensive evaluation of tissue oxygenation, tissue hemoglobin and tissue water content from a single image within 30 s.

In a porcine model of hemorrhagic shock this imaging technology enabled the reliable assessment of short-term changes in tissue and organ perfusion during shock as well as restoration of tissue perfusion in response to resuscitation [6]. In septic patients it revealed reduced tissue oxygenation in combination with increased tissue water content, which suggests that the technology could also distinguish between different types of shocks based on specific patterns [6–8]. Furthermore, HSI immediately showed the effects of induction agents in patients undergoing general anesthesia for pancreatic surgery [9].

Certified trauma centers in Germany do have a dedicated trauma resuscitation room with a dedicated trauma team for the assessment and treatment of critically injured patients. Current trauma algorithms do not include microcirculatory assessment in their primary survey, although these primary surveys are designed to detect and treat life-threatening conditions [10, 11].

To this date it is unclear whether the use of HSI is feasible in the initial treatment of a potentially critically injured patient. When implementing new diagnostic tools, there is concern that this might result in prolonged treatment times. As HSI showed promising results not only in a porcine model [6], but also in critical care [8], we assume it can have an impact on trauma patient care. To follow up on this assumption and show the feasibility in a trauma resuscitation room, we performed this randomized controlled clinical trial.

### Importance

The application of HSI could potentially provide the opportunity to detect shock objectively and directly from tissue perfusion with a single measurement.

### Goals of this investigation

The objective of this randomized controlled trial was to investigate the feasibility of acquiring a hyperspectral recording (HSR) using a dedicated camera system during the primary care in a trauma resuscitation room. Feasibility was assessed by comparing treatment times between both groups and the ability to perform hyperspectral measurements on the acquired HSR at a later timepoint. A secondary aim was to compare these hyperspectral measurements of injured patients with those of healthy volunteers.

## Material and methods

### Study design and setting

This was a randomized controlled trial conducted in a level one trauma center at a university hospital with around 748 trauma calls in 2021, of which 95 had an ISS ≥ 16. The study was approved by the Ethics Committee of Heidelberg, Germany (protocol ID S-605/2021) and registered at the German Clinical Trial Register (www.drks.de—study number DRKS00024047). Our study results are reported in accordance with the Consolidated Standards of Reporting Trials (CONSORT) guidelines [12] (Additional file 1: Tables S1 and S2). This study was conducted without external funding.

### Selection of participants

We included all adult patients (age ≥ 18 years) who were admitted to the trauma resuscitation room. Affirmation from the trauma team leader had to be obtained before the patient arrived based on the information given by the emergency medical system (EMS) physician. Patients were excluded if the cause for trauma was an attempted suicide according to the judgement from the EMS and trauma team leader, known pregnancies or if they were prisoners. All participants were recruited in the presence of at least one author.

### Healthy volunteers

Healthy volunteers were recruited from the anesthesiology department. After informed consent a HSR of a hand palm was obtained, and age and profession were recorded.

### Interventions

Patients were randomized in a 1:1 ratio between the 2 study arms before their arrival during the team timeout. Identical sealed envelopes with inclusion and exclusion criteria printed on the outside were used for the randomization process. Sequence allocation was conducted using https://www.randomizer.org. Patients allocated to the intervention group received two HSR in addition to the standard of care.

Treating physicians were blinded to the result of the hyperspectral measurements.

At the time of study inclusion participants were verbally informed of the intervention, written consent was obtained after the initial treatment from conscious patients. In unconscious patients, a legal representative was installed and asked for consent as soon as possible.

### Hyperspectral imaging

HSI was performed with the Tivita^®^ Tissue System (Diaspective Vision GmbH, Am Salzhaff, Germany). Taking an HSI is only possible with adequate exposure. The influence of ambient light was minimized by black curtains around the camera. The integrated stray light warning system indicates adequate conditions with a green dot on the screen. Camera height was then adjusted to the patients’ hand using the integrated target system with two overlapping light points. In concordance with the trauma team one patient site, whichever was better accessible, was chosen for HSI.

The technical specifications of the system have been described in previous publications [13–15]. In brief, the HSI camera captures a Red–Green–Blue-image (RGB-image) in addition to the remission spectra in a range between 500 and 1000 nm in intervals of 5 nm for every pixel (640 × 480-pixel resolution), resulting in 100 values of spectral information per pixel. These images are then used for the calculation of the following HSI parameters with their respective wavelengths [16]:

- Tissue oxygenation (StO_2_) wavelength range: 500–650 and 700–815 nm, indicated in percent (0–100%)
- Near infrared perfusion index (NIR) wavelength range: 655–735 and 825–925 nm, indicated in predefined arbitrary units (0–100)
- Tissue hemoglobin index (THI) wavelength range: 530–590 and 785–825 nm indicated in predefined arbitrary units (0–100)
- Tissue water index (TWI) wavelength range: 880–900 and 955–980 nm indicated in predefined arbitrary units (0–100).

### Hyperspectral measurements

Measurements from the HSR were performed after both images were obtained to avoid influencing the treatment. Investigator and treating physicians were blinded to the results. Measurements were performed at the palm of the respective hand and as a mean of two fingers on the same hand.

### Outcomes

The primary outcomes were the treatment duration of the primary survey and the total resuscitation room care as well as the ability to successful perform hyperspectral measurements. The duration of the primary survey was defined as the time from end of handover from the EMS crew until (1) ABCDE-assessment [10, 11] by the anesthetist, (2) standardized ultrasound by the radiologist and (3) physical examination by the trauma surgeon were completed. The total resuscitation room care was defined as the time from end of handover from the EMS crew until the patient was transferred to either the CT scan, operation room (OR), intensive care unit (ICU) or ward.

As secondary outcomes we performed two analyses for hyperspectral measurements. First, comparing the hyperspectral measurements from the injured patients with those of 26 healthy volunteers in order to assess microcirculatory impairment in injured patients.

Secondly, an analysis based on the ISS (Injury severity score) (< 16 vs. ≥ 16) to assess changes in microcirculation in major trauma patients.

### Measurements

One author (FP) acquired all HSI and performed the measurements using the TIVITA Tissue system. The first image was taken during the ongoing assessment (1–3) and the second image when the patient arrived either in the OR, ICU or at the ward. Hyperspectral measurements were StO_2_, THI, NIR and TWI as described above. Time measurements for both primary outcomes were performed by one author (FP) independently from the trauma team. In addition, we recorded demographic and clinical data as well as interventions from the EMS and trauma team during the treatment. A full list of recorded variables is listed in the Additional file 1: Table S3.

### Sample size

Treatment times vary between different systems and algorithms used. In the past, duration of the primary survey was not assessed in our resuscitation room. Therefore, it had to be defined by our study team based on our standard operating procedure. In conclusion, sample size calculation based on previous data was not possible. In concordance with our statistical department, a group size of 25 participants each was determined.

### Primary data analysis

Collection of data was done with the aid of an electronical database system (Microsoft Excel®, Microsoft Deutschland GmbH, Unterschleißheim, Germany). All analyses were performed using SPSS (Statistical Product and Services Solutions, Version 28, SPSS Inc., Chicago, IL, USA).

We performed an intention to treat analysis of the primary and secondary outcomes and since this is an exploratory analysis p-values are purely descriptive and not adjusted for multiple testing. A two-sided level of significance of 5% was considered statistically significant in the presentation of results. Demographic and baseline clinical characteristics were summarized using means and standard deviations (SD) or medians and interquartile ranges (Q1–Q3) for continuous variables and using frequencies for categorical variables. Both primary outcomes were checked for normal distribution by visual inspection and as a result analyzed by Mann–Whitney-U due to heterogeneity of the data. The effect size for the primary outcomes was calculated as the differences between pseudo medians with nonparametric 95% confidence intervals (CI) using the Hodges-Lehmann method.

Effect size in hyperspectral measurements between the intervention group and healthy volunteers was calculated as the difference in means with 95% CI using students t-test.

Missing data were not imputed.

## Results

### Patient characteristics

**Fig. 1:**
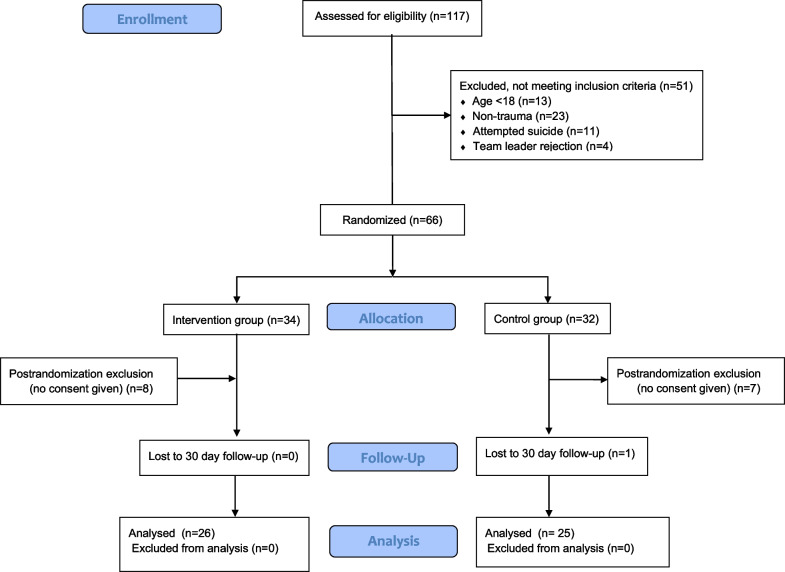
CONSORT study flow diagram [12]

Between October 26, 2021 and May 11, 2022, we screened 117 patients until we included at least 25 patients into each group. After randomization, 15 patients were excluded due to missing consent. In total we analyzed 51 patients with 26 allocated to the intervention group and 25 to control group (Fig. 1). The last 30-day follow-up was completed on June, 13 2022. Patients’ baseline characteristics, the underlying trauma mechanism, vital parameters on arrival and diagnostics performed are presented in Table 1. The average age was 50 years (SD 16) and 44 years (SD 18), with 23% and 28% of the patients being women in the intervention- and control group, respectively. Overall, most reasons for admittance were road traffic accidents (62.7%) and falls from height ≥ 3 m (23.5%). ISS, vital parameters and blood gas analysis parameters on admission were similar in both groups. All patients had an ultrasound performed, most (92.3% and 84.0%) had an immediate CT scan.

**Table 1:** Patients’ baseline characteristics

	Intervention group (n = 26)	Control group (n = 25)
Age, [y] mean (SD)	50 (16)	44 (18)
Female sex, n (%)	6 (23%)	7 (28%)
ISS ≥ 16, n (%)	2 (8%)	2 (8%)
*Trauma mechanism, n (%)*		
Fall from height ≤ 3 m	–	2 (8%)
Fall from height > 3 m	9 (34.6%)	3 (12%)
Car accident ≤ 100 km/h	6 (23.1%)	7 (28%)
Car accident > 100 km/h	6 (23.1%)	5 (20%)
Motorcycle accident	3 (11.5%)	5 (20%)
Penetrating trauma	–	1 (4%)
Other	2 (7.7%)	1 (4%)
*Vital parameters on admission, mean (SD)*		
SpO_2_ [%]	98 (1)	98 (2)
Heart rate [1/min]	79 (13)	86 (14)
Capillary refill time [s]	2 (1)	2 (1)
Blood pressure, systolic [mmHg]	138 (18)	144 (15)
Temperature [°C]	36.8 (0.5)	36.7 (0.5)
GCS	15 (0)	15 (0)
ISS	7 (5)	5 (5)
*Blood gas analysis on admission, mean (SD)*		
pH	7.36 (0.05)	7.38 (0.06)
Base excess [mmol/l]	− 1.28 (2.52)	− 1.25 (2.18)
Hemoglobin [g/dl]	13.53 (1.83)	13.65 (0.94)
Lactate [mg/dl]	15.67 (6.11)	16.61 (6.77)
*Diagnostics, n (%)*		
Ultrasound	26 (100%)	25 (100%)
X-ray	5 (19.2%)	3 (12%)
CT scan	24 (92.3%)	21 (84%)

*SD* standard deviation; *GCS* Glasgow Coma Scale; *ISS* injury severity score

Normal ranges: pH 7.37–7.45, Base Excess −2 to +3, Hemoglobin 12 to 15 and Lactate < 16

### Outcomes

#### Primary outcomes—treatment times

We found a significant difference in the median duration of the primary survey with 03:59 min (Q1 to Q3 03:29 to 04:35) in the intervention group compared to 03:22 min (Q1 to Q3 03:00 to 03:51) in the control group (Difference -00:37 [95% CI -01:06 to -00:12]). There was no statistical significance between both groups in the total resuscitation room care (Table 2). We were able to perform hyperspectral measurements from all acquired images.

**Table 2:** Primary outcomes

	Intervention group (n = 26)	Control group (n = 25)	Estimated difference (95% CI)
Primary survey [min], median (Q1–Q3)	03:59 (03:29–04:35)	03:22 (03:00–03:51)	−00:37 (−01:06 to −00:12)
Resuscitation room care [min], median (Q1–Q3)	08:00 (07:00–12:00)	10:00 (07:00–13:00)	01:00 (−01:00 to 03:00)

*CI* confidence interval

#### Secondary outcomes—hyperspectral image measurements

**Fig. 2:**
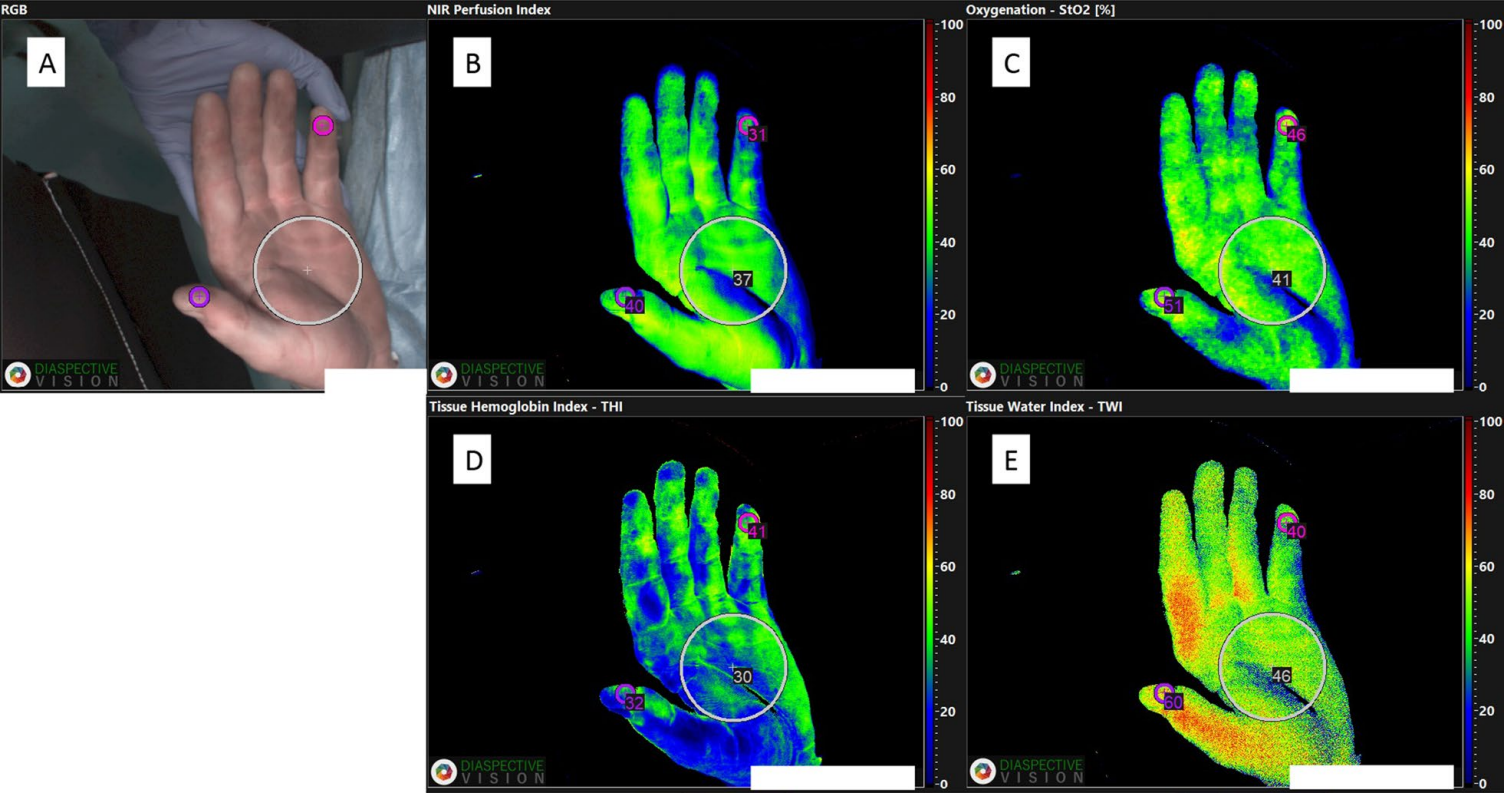
**A**–**E** Hyperspectral image during the initial resuscitation room care of the left hand of patient #2 with measurements of the palm and two fingertips (D1 and D5). All values are color coded. Red/yellow indicate a higher value (50–100), whereas green/blue indicate a lower value (0–50). The larger circle indicates the area of the palm. StO_2_ **C** is presented as percentage, NIR (**B**), THI (**D**) and TWI (**E**) are presented as numeric values

**Fig. 3:**
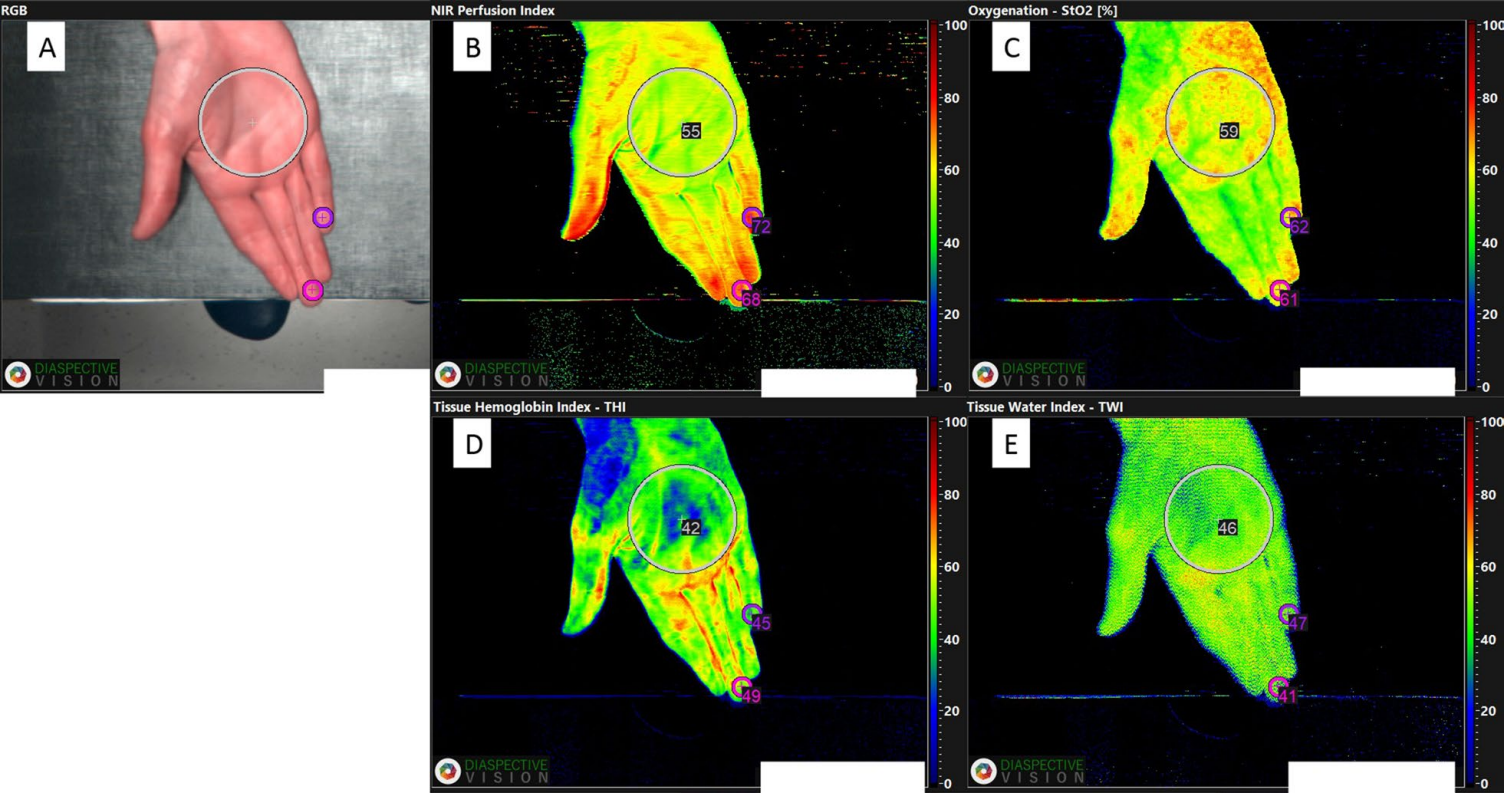
**A**–**E** Hyperspectral image of the right hand of a healthy volunteer with measurements of the palm and two fingertips (D3 and D5)

We compared the initial HSI measurement (Measurement 1, Table 3) with the control group, as this was obtained before in-hospital treatment was started. The measured NIR perfusion of the palm and fingertips were significantly reduced with 51.5 (SD 9.8) versus 57.7 (SD 4.6) and 57.9 (SD 12.9) versus 71.5 (SD 6.9) with differences of − 6.24 (95% CI -10.61 to − 1.86) and -13.61 (95% CI − 19.48 to − 7.75) in injured patients compared to healthy volunteers, respectively. StO2 of the palm was significantly lower in the intervention group with 55.9% (SD 10.1) compared to 65.8% (SD 7.9) with a difference of − 9.9 (95% CI − 15.08 to − 4.71). Additionally, TWI of both the palm and fingertips were significantly higher in injured patients. The full list of HSI measurements is presented in Table 3. Figures 2 and 3 demonstrates an HSR with measurements of the hand palm.

**Table 3:** Secondary outcomes

	Intervention group (n = 26)		Healthy volunteers (n = 26)	Difference (95% CI)
	Measurement 1	Measurement 2		
NIR palm	51.48 (9.84)	53.73 (9.27)	57.72 (4.61)	−6.24 (−10.61 to −1.86)
NIR fingertip	57.86 (12.93)	62.63 (13.37)	71.48 (6.86)	−13.61 (−19.48 to −7.75)
StO_2_ palm	55.90 (10.15)	60.74 (9.40)	65.80 (7.94)	−9.9 (−15.08 to −4.71)
StO_2_ fingertip	66.98 (11.62)	69.96 (10.22)	72.42 (8.17)	−5.44 (−11.11 to –0.24)
TWI palm	52.08 (12.27)	50.42 (6.57)	43.96 (6.76)	8.12 (2.48 to 13.75)
TWI fingertip	54.58 (12.18)	50.56 (7.51)	37.08 (10.91)	17.50 (10.99 to 24.02)
THI palm	31.42 (14.90)	29.58 (7.90)	29.88 (6.72)	1.54 (−5.03 to 8.11)
THI fingertip	46.04 (12.30)	43.69 (7.47)	45.74 (8.18)	0.30 (−5.60 to 6.20)

HSI Measurements are presented as mean with standard deviation as index (NIR, TWI, THI) or percentages with standard deviation (StO_2_). Difference was calculated between Measurement 1 and Healthy volunteers; *CI* confidence interval; *NIR* near infrared perfusion index; StO_2_ tissue oxygen saturation; *TWI* tissue water index; *THI* tissue hemoglobin index

In addition, we compared HSI measurements between patients with an ISS ≥ 16 compared to those with < 16 and found no significant difference for any HSI parameter (Additional file 1: Table S4).

### Patient outcomes

30 days follow-up was conducted either by e-mail or a phone call. We lost one patient in the control group to 30 days follow-up. In the control group, 5 (21.5%) patients had to be admitted to the ICU and 3 (12.5%) patients underwent surgery immediately. Another 3 (12.5%) patients were discharged on the same day. In the intervention group, 8 (30.8%) patients were admitted to the ICU and 5 (19.2%) went to the OR. No patient was discharged on the same day. In both groups, all patients were alive throughout the 30 days follow up-period.

## Limitations

Several limitations should be considered for the interpretation of the results. First, it was only conducted at a single trauma center, limiting its external validity. Secondly, our study sample size was too small to analyze subgroups with severe trauma or shock. This is reflected in the non-significance when assessing HSI measurements in patients with an ISS < 16 and ≥ 16. However, this is, to the best of our knowledge, the first study that shows the feasibility of HSI in the trauma resuscitation room. Additionally, the study was not designed to assess any differences between patients with minor and major trauma. Further studies are therefore necessary to investigate the use of HSI for shock recognition in trauma care. Third, we had to exclude four patients due to rejection of the trauma team leader. One of those patients suffered a gunshot wound, another patient a traumatic cardiac arrest. Both patients were major traumas and could have potentially contributed to the subgroup analysis according to ISS. As those patients are scarce in our trauma center, including them in our analysis would have provided a better insight into microcirculatory alterations in severely injured patients. Furthermore, during primary care of patients suffering minor trauma an additional diagnostic tool is more likely to be accepted by the trauma team.

## Discussion

This randomized controlled trial demonstrated the feasibility of implementing HSI as a novel diagnostic tool of microcirculatory diagnostics in our trauma resuscitation room. The use of HSI led to a statistically significant prolongation of the primary survey. However, the total duration of resuscitation room care was not influenced by the additional measurement. We were able to perform hyperspectral measurements from all HSR taken during the primary survey and after the initial treatment was completed. Therefore, we conclude that there was no clinically relevant influence on trauma care. Interestingly, even patients with a minor trauma tend to have a reduced microcirculatory tissue oxygenation when compared to healthy volunteers. Although hemodynamic and laboratory parameters, such as blood pressure, CRT and lactate were within the normal clinical ranges. This could possibly be explained by an increased level of stress, leading to higher endogenous catecholamine release in consequence to trauma. Resulting in peripheral vasoconstriction and consequently a reduced microcirculatory perfusion. Elevated levels of serum epinephrine and norepinephrine were found in patients with traumatic brain injury [17] and hemorrhagic shock after trauma [18]. Even in patients undergoing general anesthesia, the immediate effects of induction agents, leading to a significant increase of tissue oxygenation, perfusion index and tissue water index have been demonstrated [9].

Until now, there are no defined standard values for the parameters derived from HSI for humans. On the other hand, normal ranges have been demonstrated in a porcine model not only for the tissue, but for 20 organs [19]. The observed difference from trauma patients to healthy volunteers suggests that lower values should be expected in this patient group. Further studies should investigate the comparison of hemodynamically stable patients to patients with shock after trauma to allow the detection of critical patients.

Unfortunately, as this study was only designed to proof feasibility, it lacks the possibility to perform a subgroup analysis based on the presence or absence of hemodynamic instability. Nevertheless, hyperspectral imaging proofed to be a reliable tool in detecting microcirculatory alterations in hemorrhagic shock in a porcine model [6]. Reduced tissue perfusion is a key pathological finding in shock. In critically ill patients, microcirculatory dysfunction is associated with an increased risk of organ dysfunction, morbidity and mortality [20]. Given the 30 days follow up in the present study, those changes in microcirculation had no impact on the survival rate or morbidity after the incidence. Due to the low rate of complications and small sample size we were not able to assess the link between microcirculation and morbidity as well as survival rate in trauma patients.

In retrospect, we would obtain the skin temperature of the respective hand. Allowing us to determine the influence of temperature on the hyperspectral measurements.

To further assess and understand microcirculatory changes and the link to morbidity and mortality in patients with major trauma, we plan on conducting a prospective observational trial including a larger cohort with any type of trauma.

In summary, the use of HSI during the primary survey in trauma did not delay existing treatment protocols. Furthermore, this novel diagnostic tool can provide valuable information on the microcirculatory state and could potentially facilitate recognition of critical patients.

## Supplementary Information

**Additional file 1**. Supporting Information.

## Data Availability

The full datasets used during the current study are available from the corresponding author on reasonable request.

## Abbreviations

CI: Confidence interval
CRT: Capillary refill time
EMS: Emergency medical system
GCS: Glasgow Coma Scale
HSI: Hyperspectral imaging
HSR: Hyperspectral recordings
ISS: Injury severity score
NIR: Near infrared perfusion index
SD: Standard deviation
StO_2_: Tissue oxygen saturation
THI: Tissue hemoglobin index
TWI: Tissue water index
