# Supplementary material for: Implementation of hyperspectral imaging in a trauma resuscitation room: a randomized controlled trial
Source: Scand J Trauma Resusc Emerg Med. 2022 Dec 9;30:66. doi: 10.1186/s13049-022-01057-7 (PMC9733002; doi:10.1186/s13049-022-01057-7)
Supplement: Supplementary file 1 — Additional file 1. Supporting Information. [file 13049_2022_1057_MOESM1_ESM.docx]

**Additional file 1**

**Implementation of hyperspectral imaging in a trauma resuscitation room – a randomized controlled clinical trial**

Table of contents

[S1 – CONSORT abstract checklist 2](#_Toc111095593)

[S2 - CONSORT checklist 3](#_Toc111095594)

[S3 - List of variables 7](#_Toc111095595)

[S4 - HSR Measurements for subgroups 9](#_Toc111095596)

# Table S1 – CONSORT abstract checklist

| **Item** | **Description** | **Reported on line number** |
| --- | --- | --- |
| Title | Identification of the study as randomized | 1 |
| Authors * | Contact details for the corresponding author |  |
| Trial design | Description of the trial design (e.g. parallel, cluster, non-inferiority) | 28-30 |
| Methods |  |  |
| Participants | Eligibility criteria for participants and the settings where the data were collected | 29-30 |
| Interventions | Interventions intended for each group | 31-32 |
| Objective | Specific objective or hypothesis | 33-36 |
| Outcome | Clearly defined primary outcome for this report | 33-36 |
| Randomization | How participants were allocated to interventions | 30 |
| Blinding (masking) | Whether or not participants, care givers, and those assessing the outcomes were blinded to group assignment | 38-39 |
| Results |  |  |
| Numbers randomized | Number of participants randomized to each group | 41 |
| Recruitment | Trial status | 41 |
| Numbers analysed | Number of participants analysed in each group | 41 |
| Outcome | For the primary outcome, a result for each group and the estimated effect size and its precision | 43-45 |
| Harms | Important adverse events or side effects | N/A |
| Conclusions | General interpretation of the results | 50-51 |
| Trial registration | Registration number and name of trial register | 53 |
| Funding | Source of funding | 55 |

# Table S2 - CONSORT checklist


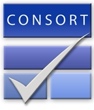
CONSORT 2010 checklist of information to include when reporting a randomised trial

| Section/Topic | Item No | Checklist item | Reported on page No |
| --- | --- | --- | --- |
| Title and abstract | | | |
|  | 1a | Identification as a randomised trial in the title | Title Page |
|  | 1b | Structured summary of trial design, methods, results, and conclusions (for specific guidance see CONSORT for abstracts) | Abstract |
| Introduction | | | |
| Background and objectives | 2a | Scientific background and explanation of rationale | Introduction/ Background |
|  | 2b | Specific objectives or hypotheses | Introduction/ Goals of this investigation |
| Methods | | | |
| Trial design | 3a | Description of trial design (such as parallel, factorial) including allocation ratio | Material and Methods/ Study Design and setting |
|  | 3b | Important changes to methods after trial commencement (such as eligibility criteria), with reasons | N/A |
| Participants | 4a | Eligibility criteria for participants | Material and Methods/ Selection of Participants |
|  | 4b | Settings and locations where the data were collected | Material and Methods/ Study Design and setting |
| Interventions | 5 | The interventions for each group with sufficient details to allow replication, including how and when they were actually administered | Material and Methods/ Interventions |
| Outcomes | 6a | Completely defined pre-specified primary and secondary outcome measures, including how and when they were assessed | Material and Methods/ Outcomes |
|  | 6b | Any changes to trial outcomes after the trial commenced, with reasons | N/A |
| Sample size | 7a | How sample size was determined | Material and Methods/ Sample size |
|  | 7b | When applicable, explanation of any interim analyses and stopping guidelines | N/A |
| Randomisation: |  |  |  |
| Sequence generation | 8a | Method used to generate the random allocation sequence | Material and Methods/ Interventions |
|  | 8b | Type of randomisation; details of any restriction (such as blocking and block size) | Material and Methods/ Interventions |
| Allocation concealment mechanism | 9 | Mechanism used to implement the random allocation sequence (such as sequentially numbered containers), describing any steps taken to conceal the sequence until interventions were assigned | Material and Methods/ Interventions |
| Implementation | 10 | Who generated the random allocation sequence, who enrolled participants, and who assigned participants to interventions | Material and Methods/ Interventions |
| Blinding | 11a | If done, who was blinded after assignment to interventions (for example, participants, care providers, those assessing outcomes) and how | Material and Methods/ Hyperspectral measurements; Material and Methods/ Primary Data Analysis |
|  | 11b | If relevant, description of the similarity of interventions | N/A |
| Statistical methods | 12a | Statistical methods used to compare groups for primary and secondary outcomes | Material and Methods/ Primary Data Analysis |
|  | 12b | Methods for additional analyses, such as subgroup analyses and adjusted analyses | Material and Methods/ Primary Data Analysis |
| Results | | | |
| Participant flow (a diagram is strongly recommended) | 13a | For each group, the numbers of participants who were randomly assigned, received intended treatment, and were analysed for the primary outcome | Results/ Patient Characteristics and Figure 1 |
|  | 13b | For each group, losses and exclusions after randomisation, together with reasons | Results/ Figure 1 |
| Recruitment | 14a | Dates defining the periods of recruitment and follow-up | Results/ Patient Characteristics |
|  | 14b | Why the trial ended or was stopped | Results/ Patient Characteristics |
| Baseline data | 15 | A table showing baseline demographic and clinical characteristics for each group | Results/ Table 1 |
| Numbers analysed | 16 | For each group, number of participants (denominator) included in each analysis and whether the analysis was by original assigned groups | Results/ Patient Characteristics |
| Outcomes and estimation | 17a | For each primary and secondary outcome, results for each group, and the estimated effect size and its precision (such as 95% confidence interval) | Outcomes/Primary Outcomes – Treatment times; Outcomes/ Table 2; Outcomes/ Secondary Outcomes – Hyperspectral image measurements; Outcomes/ Table 3 |
|  | 17b | For binary outcomes, presentation of both absolute and relative effect sizes is recommended | N/A |
| Ancillary analyses | 18 | Results of any other analyses performed, including subgroup analyses and adjusted analyses, distinguishing pre-specified from exploratory | N/A |
| Harms | 19 | All important harms or unintended effects in each group (for specific guidance see CONSORT for harms) | N/A |
| Discussion | | | |
| Limitations | 20 | Trial limitations, addressing sources of potential bias, imprecision, and, if relevant, multiplicity of analyses | Limitations |
| Generalisability | 21 | Generalisability (external validity, applicability) of the trial findings | Limitiations |
| Interpretation | 22 | Interpretation consistent with results, balancing benefits and harms, and considering other relevant evidence | Discussion |
| Other information | | |  |
| Registration | 23 | Registration number and name of trial registry | Material and Methods/ Study Design and setting |
| Protocol | 24 | Where the full trial protocol can be accessed, if available | Material and Methods/ Study Design and setting |
| Funding | 25 | Sources of funding and other support (such as supply of drugs), role of funders | Material and Methods/ Study Design and setting |

# Table S3 - List of variables

Patient history

| **Parameter** | **Unit of measurement** |
| --- | --- |
| Age | Years |
| Sex | Male/female |
| Weight | Kilogram |
| Height | Centimeter |
| Pre-existing conditions | List |
| Medication | List |
| Allergies | List |
| Alcohol, Smoking | List |
| Previous operations | List |

Current Incidence

| **Parameters** | **Unit of measurement** |
| --- | --- |
| Time of incidence | HH:MM |
| Trauma mechanism | List |
| Injuries | List |
| Vital signs on admission (Pulse, Blood Pressure, Temperature, Capillary refill time, SpO2, Respiratory rate, Blood sugar, ECG, GCS) | Measurements were standard units for the respective parameter |
| Injury severity score | Scale 0-75 |
| Time of arrival | HH:MM |

Treatment

| **Parameters** | **Unit of measurement** |
| --- | --- |
| Pharmacological Treatment  Noradrenaline  Adrenaline  Vasopressin  Dobutamine  Sufentanil  Fentanyl  Piritramide  Midazolam  Esketamine  Propofol  Rocuronium | µg  µg  IE/kg/min  mg/kg/min  µg  µg  mg  mg  mg  mg  mg |
| Fluids  Crystalloids  Colloids  Red blood cell transfusion  Plasma transfusion  Fibrinogen transfusion | ml  ml  ml  ml  ml |
| Needle decompression | Yes / No |
| Pelvic sling | Yes / No |
| Tourniquet | Yes / No |
| Thoracostomy | Yes / No |
| REBOA | Yes / No |
| Thoracotomy | Yes / No |
| Intubation | Yes / No |

# Table S4 - HSR Measurements for subgroups

ISS <16 vs. ISS ≥16

|  | **ISS <16 (n = 24)** | **ISS ≥16 (n = 2)** | **Difference (95% CI)** |
| --- | --- | --- | --- |
| NIR Palm | 51.89 (9.69) | 46.75 (14.49) | 5.14 (-10.03 to 20.31) |
| NIR Finger | 57.14 (13.04) | 66.50 (10.60) | -9.35 (-29.02 to 10.31) |
| StO_2_ Palm | 56.41 (10.33) | 50.00 (7.07) | 6.41 (-9.15 to 21.98) |
| StO_2_ Finger | 67.18 (11.61) | 64.50 (16.26) | 2.68 (-15.30 to 20.67) |
| TWI Palm | 51.67 (12.72) | 56.75 (1.06) | -5.07 (-24.06 to 13.91) |
| TWI Finger | 54.47 (12.57) | 56.00 (8.48) | -1.53 (-20.41 to 17.35) |
| THI Palm | 30.45 (13.26) | 42.50 (34.64) | -12.04 (-34.68 to 10.59) |
| THI Finger | 45.75 (12.32) | 49.50 (16.26) | -3.75 (-22.76 to 15.26) |

*Caption: HSR Measurements are presented as mean with standard deviation as index (NIR, TWI, THI) or percentages (StO_2_).*

*CI = Confidence interval; NIR = Near infrared perfusion index; StO_2_ = Tissue oxygen saturation; TWI = Tissue water index; THI = Tissue hemoglobin index*
